# Thr92Ala Polymorphism of Human Type 2 Deiodinase Gene (hD2) Affects the Development of Graves' Disease, Treatment Efficiency, and Rate of Remission

**DOI:** 10.1155/2012/340542

**Published:** 2012-11-12

**Authors:** Babenko Alina, Popkova Daria, Freylihman Olga, Solncev Vladislav, Kostareva Anna, Grineva Elena

**Affiliations:** ^1^Institute of Endocrinology, Almazov Federal Heart, Blood and Endocrinology Centre, 2 Akkuratova Street, Saint-Petersburg 197541, Russia; ^2^Institute of Molecular Biology and Genetics, Almazov Federal Heart, Blood and Endocrinology Centre, 2 Akkuratova Street, Saint-Petersburg 197541, Russia; ^3^Department of Mathematical Modeling, Almazov Federal Heart, Blood and Endocrinology Centre, 2 Akkuratova street, Saint-Petersburg 197541, Russia

## Abstract

Clinical symptoms vary in thyrotoxicosis, and severity of these depends on many factors. Over the last years, impact of genetic factors upon the development and clinical significance of thyrotoxic symptoms became evident. It is known that a production of T3 in various tissues is limited by deiodinase 2 (D2). Recent studies revealed that certain single nucleotide polymorphisms (including threonine (Thr) to alanine (Ala) replacement in D2 gene codon 92, D2 Thr92Ala) affect T3 levels in tissues and in serum. Individuals with Ala92Ala genotype have lower D2 activity in tissues, compared with that in individuals with other genotypes. In our study, we have assessed an association of D2 Thr92Ala polymorphism with (1) frequency of disease development, (2) severity of clinical symptoms of thyrotoxicosis, and (3) rate of remissions, in Graves' disease patients.

## 1. Introduction

Over the last years, much attention has been paid to the emerging concept of a “personalized therapy.” This recently developed approach, in particular, presumes using the information on patient's genotypes for the optimization of one's therapy. Analysis of genetic predisposition to sulfonylurea drug response in diabetes patients (Ser1369Ala variant in ABCC8 gene) serves as an example of the personalized therapy [1]. Ever increased introduction of genetic tests to the clinical laboratory promises developing a new therapeutic strategy—the personalized therapy of various diseases. In the current study, we have evaluated human type 2 deiodinase gene (hD2) polymorphism Thr92Ala as a potential genetic predictor of response to thyrostatic therapy in Graves' disease (GD). 

Deiodinases are the selenoenzymes regulating the transformation of thyroxin (T4) into triiodothyronine (T3) [2–4]. Type 1 deiodinase (D1) is expressed and synthesized in liver, kidney, and thyroid gland [2, 3] and is responsible for the levels of circulating T3 hormone [2–6]. Type 2 deiodinase (D2) enables T3 production in central nervous system, pituitary gland, brown adipose tissue, cardiac and skeletal muscle, and placenta [2–4]; it is expressed on lower levels in liver and kidney [2, 3]. Thus, D2 plays the key role in local tissue T3 production [6–11]. According to the published data, type 2 deiodinase activities increase manyfold in some tissues in Graves' disease patients [10]. 

Recent studies showed that polymorphisms of some deiodinase genes affect the production of thyroid hormones: human D2 gene, threonine (Thr) to alanine (Ala) replacement in codon 92 (D2 Thr92Ala) among them [2, 5, 12]. Ala92Ala homozygous subjects demonstrate lower D2 tissue activity compared to Ala/Thr heterozygous and Thr/Thr homozygous subjects [12, 13]. Thus, Ala/Ala homozygous subjects have lower T3 effects in tissues with high D2 gene expression [2–8]. As Ala92Ala genotype association with insulin resistance and arterial hypertensions is well established [12, 14, 15], this polymorphism is suggested to influence clinical manifestations and the severity of heart damage in patients with thyrotoxicosis.

Our previous study aimed investigating the impact of D2 Thr92Ala polymorphism on the clinical course, laboratory, and EchoCG parameters in patients with Graves' disease [16]. We have identified negative correlation between Ala92Ala genotype and thyroid volume, and between the former and T3/T4 ratio. Thr92Thr genotype was associated with a risk of development of eccentric left ventricular hypertrophy [16]. In the current study, we have investigated (1) frequency of disease development, (2) severity of clinical symptoms of thyrotoxicosis, and (3) rate of remissions, in Graves' disease patients with various genotypes of type 2 deiodinase Thr92Ala polymorphism.

## 2. Patients and Methods

### 2.1. Patients

 All patients with Graves' disease, either hospitalized or from outpatient department of the Almazov Federal Centre during year 2005–2010, were assessed for the following inclusion/exclusion criteria.

Inclusion criteria:

1. age 20–55;
2. established thyrotoxicosis associated with Graves' disease at the primary examination;
3. consent of patient for participation in this study;
4. the high quality of EchoCG images was required for better evaluation of heart structure and function.

Exclusion criteria:

1. concomitant cardiovascular diseases that can result in fixed abnormal changes of EchoCG parameters (heart ischemic disease, hypertension, valvular disease, nonthyrotoxic cardiomiopathy, heart failure, diabetes mellitus, obstructive lung disease, and nonthyreotoxic arrhythmias);
2. diseases have contraindications for long thyrostatic therapy (increase ALT or AST more 5-point normal range, hepatic or renal failure, intolerance thioamides);
3. intoxication (alcohol, toxicomania);
4. pregnancy or plane of pregnancy.

Among 250 screened patients 180 patients met the inclusion/exclusion criteria with 1- to 15-year-long history of Graves' disease; aged 18 to 54 years, without concomitant diseases, were included in the study (Table 1). The diagnosis of GD was confirmed by the presence of thyrotoxicosis, diffuse hyper functional goiter, and of autoantibodies to thyroid stimulating hormone (TSH) receptor and/or increased radioactive iodine uptake, at the moment of examination or in anamnesis.

Presence of overt thyrotoxicosis was based on levels of free T3 and free T4 above normal range and level of TSH lower than 0.1 mU/L.

Presence of subclinical thyrotoxicosis was based on level TSH lower than 0.1 mU/L with normal levels of free T3 and free T4. 

For the start of Graves' disease was accepted the time of first registration of clinical signs of thyrotoxicosis with laboratory criteria (levels of free T3 and free T4 above normal range and level of TSH lower than 0.1 mU/L).

This study was approved by the local Ethical Committee of Almazov Federal Centre. 

All patient were examined prior to the beginning of the thyreostatic therapy. All patients were treated by thioamides (mercasolil) in dose 30 mg and dose of thioamides was decreased to 10 mg (supporting dose) after restoration of euthyroidism (about 2-3 month treatment). In followup were 95 patients included; other patients were inaccessible to objective and laboratory inspection and were dropped from followup. These 95 patients were reexamined 1 and 2 years following the beginning of the therapy. In all followup visits, GD patients were checked for remission, according to the criteria associated with its high probability: normalization of thyroid volume (TV) and thyroid blood flow (TBF); absence of antibodies to TSH receptor; normalization of fT3, fT4, and TSH levels [17]. 

Remission was defined as the the time of registration of remission' criteria, but the patient was considered to reach remission only if remission fact was confirmed by the preservation of proof euthyreoidism in a year after cancellation of thyreostatic therapy [17]. In case of repeated thyreotoxicosis after the cancellation of thyrostatics the relapse was diagnosed.

A group of 135 age- and gender-matched euthyroid blood donors living in the same region constituted the control group. 

### 2.2. Methods

Free thyroid hormones and antibody serum levels were measured by immune-enzyme assay using ACCESS 2 analyzer (Beckman Coulter, USA) and immunochemical test systems (UNICEL DXI 800 ACCESS, Beckman Coulter): free triiodothyronine (fT3, the normal range is 4.0–8.0 pmol/L), free thyroxin (fT4, the normal range is 10–25 pmol/L), TSH (the normal range is 0.25–3.5 mU/L), thyroperoxidase antibodies (TPOab, the normal range = 0–30 IU/mL), and antibodies to TSH receptor (rcTSHab, the normal range is <1.0 IU/L).

 Ultrasound examination of thyroid gland was performed using ADR-2002 device with high-resolution 7.5 MHz sensors. Thyroid volume (TV) was calculated using the following method: (i) TV_lobe_ = 0.5 × *l* × *h* × *m* (*l*, lengths; *h*, height; *m*, width of thyroid lobe); (ii) volume of 2 thyroid lobes (left and right) is summarized as TV = TV_right  lobe_ + TV_left  lobe_. Normal TV range for women and men is ≤18 cm^3^ and ≤25 cm^3^, respectively. 

DNA was isolated from 200 *μ*L volume of the peripheral blood by phenol-chloroform extraction. Genotyping was performed by polymerase chain reaction/restriction fragment length polymorphism (PCR-RFLP) method using the following primers: RV: 5′-CTCAGGGCTGGCAAAGTCAAG; FW: 5′-CCACACTCTATTAGAGCCAATTG. Cycling conditions were as follows: the initial cycle of 30 seconds at 95°C, 30 seconds at 55°C, and 1 min at 72°C; 30 cycles and a final extension at 72°C for 5 minutes. 

D2 genotype was identified by the endonuclease *Bsgl*-induced restriction (NEB, UK) of the PCR products at 37°C over night at the recommended conditions. Restriction fragments were resolved in a standard 1.5% agarose gel. To verify the genotyping method and the results the identification of genotypes from 30 random DNA samples was performed by direct sequencing. 

### 2.3. Statistical Analysis

The results were expressed as frequencies, mean ± S.D., or median and percentiles 25–75 (P25–75). Allelic frequencies were determined by gene counting, and deviations from the Hardy-Weinberg equilibrium were verified using an *χ* ^2^ test. Clinical and laboratory data were addressed using *χ* ^2^ test, unpaired Student's *t*-test, Mann-Whitney *U*-test, ANOVA, Kruskal-Wallis *H*-test, Fisher exact test, or multiple logistic regression analysis, as appropriate. A two-tailed *P* < 0.05 was considered statistically significant, and all analyses were performed by STATISTICA 6.0 software package (StatSoft Inc., USA). 

## 3. Results and Discussion

 The baseline characteristics of age- and gender-matched groups of patients with Graves' disease (180 subjects) and controls (135 subjects) are presented in Table 1. A-allele (92Ala) frequency in patients with Graves' disease was 0.240, while it was 0.320 in the control group (*P* < 0.0001). In the Graves' disease group, 106 subjects (62.3%) were homozygous for 92Thr allele (TT genotype), 60 (33.3%) were heterozygous (TA), and 14 (7.8%) were homozygous for 92Ala-allele (AA). In the control group, 53 individuals (39.3%) had TT genotype, 79 (58.5%) were heterozygous (TA), and 3 (2.2%) had AA genotype. For the group of patients with Graves' disease, the genotypes were in Hardy-Weinberg equilibrium (*P* = 0.52; expected frequencies: TT = 57.2%, 36.9%, AA = 6.0%). However, in the control group, allele frequencies were in disequilibrium (*P* = 0.0002) (expected frequencies: TT = 46.9%, AT = 43.2%, AA = 9.9%), which can be partly explained by the low number of included subjects as well as the low frequency of A-allele.

The frequency of homozygotes for the T-allele (92Thr) was significantly higher in the group of patients with Graves' disease comparing to the control group (58.9% versus 39.3% resp.; *P* < 0.0001). Therefore, odds ratio (OR) was 2.20 (95% CI 1.40–3.47, *P* = 0.0007) for the TT genotype in Graves' disease patients. On the contrary, AT genotype produces inverse relationship, with a chance to develop disease being essentially lower in this group; OR = 0.36 (95% CI 0.24–0.57, *P* < 0.0001). The frequency of the minor A-allele (92Ala) was significantly more low in the group of patients with Graves' disease comparing to the control group (0.240 versus 0.320, resp.; *P* < 0.0001). 

Characteristics of patients with Graves' disease (*N* = 180) having different genotypes are presented in Table 2. There was no significant age difference between the patients with different genotypes. It is worth noting that no males with AA genotype were identified in the study.

Characteristics of patients with Graves' disease having different genotypes were compared. Thyroid volume (TV) and fT3 level were significantly higher in patients with TT genotype, comparing to the patients with other genotypes (*P* < 0.01 and *P* < 0.001, resp.). Heart rate in patients with TT and TA genotypes (99.8 ± 3.01 and 96.7 ± 2.80 beats/min, correspondingly) was significantly higher, comparing to that in A-allele homozygous patients (AA genotype) (87.2 ± 5.30 beats/min, *P* < 0.01). There was no correlation of blood pressure parameters to the genotype. Notably, the duration of the disease was significantly longer in T-allele homozygous patients (TT genotype) (*P* = 0.007) (Table 3). 

Negative correlation between disease relapse frequency and AA genotype was identified (*P* < 0.01) (Table 4). We therefore suggest that absence of A-allele predicts high rate of Graves' disease recurrence. We have addressed this hypothesis by performing analysis of the efficiency of conservative therapy in thyrotoxicosis patients with various D2 Thr92Ala genotypes. Dynamic followup was performed for 95 patients during 2 years of conservative treatment (repeated visits every 2-3 months), including the monitoring of hormone level and evaluation of remission. Criteria associated with high probability of remission were as follows: normalization of a thyroid gland volume and thyroid blood flow (TBF), reduction of TSH receptor antibody levels to the normal range, and euthyreoidism on the minimal dose of thyrostatics (10 mg mercasolil). Subsequently, followup was continued for those patients who had reached remissions and did not continue therapy during a year to prove the remission. We have found that frequency of AA genotype in the group of patients with euthyreoidism on the supporting dose of thyreostatics through the treatment was twice as high as that among patients with recurrence of thyrotoxicosis on the supporting dose of mercasolil (10 mg/d), 8.3% and 3.0%, respectively (*P* < 0.01). Distribution of the genotype frequencies within groups of Graves' disease patients is presented in Table 4. Most notably, these values correspond to OR = 7.90 (95% CI 2.0–32.3, *P* = 0.002) for T-allele in Graves' disease patients undergoing remission. 

## 4. Conclusion

As functional activity of type 2 deiodinase is associated with polymorphism of D2 gene at 92 position [2], carriers of Ala92Ala genotype have lower activity of the enzyme and, accordingly, less active education of T3 in tissues. The key result of our study is the identification of the association of Ala92Ala genotype with high frequency of remission in Graves' disease patients (7.9-fold more frequent achievement of steady remission as a result of conservative therapy). The carriers of A-allele have milder thyrotoxicosis (i.e., lower levels of circulating thyroid hormones, low T3/T4 ratio, high level of TPO antibodies, and lower heart rate) that possibly facilitates achievement of remission in the subgroup of patients carrying A-allele. Data generated in this study suggest that AA genotype Ala92Thr polymorphism of D2 gene is protective, in regard to (1) the frequency of Graves' disease development, (2) severity of disease, and (3) rate of remissions in the patients.

Results of the present study provide further implications of the genetic factors in the variations of response to conservative therapy at thyrotoxicosis in Graves' disease patients. 

## Figures and Tables

**Table 1:** Characteristics of the study groups.

	Graves' disease (*n* = 180)	Healthy controls (*n* = 135)
Age (years)	42.7 ± 0.72	46.2 ± 8.8
Sex (male/female)	36/144	24/110
Heart rate (bpm)	98.7 ± 1.44	72.6 ± 0.87
Blood pressure (mmHg)	130.5 ± 1.17/77.1 ± 0.67	125.3 ± 0.97/72.4 ± 0.71

**Table 2:** Characteristics of patients with Graves' disease, type 2 deiodinase polymorphism genotypes.

	D2 gene genotypes		
	TT (*n* = 106)	AT (*n* = 60)	AA (*n* = 14)
Genotype frequency	0.589	0.333	0.078
Age (years)	42.9 ± 0.99	42.2 ± 1.16	40.9 ± 3.10
Sex (male/female)	13/93	11/49	0/12
TV (cm^3^)	33.5 ± 2.27	27.2 ± 2.55*	24.6 ± 2.35*
fT3 (pmol/L)	15.99 ± 1.55	11.56 ± 1.93*	10.7 ± 1.85*
fT4 (pmol/L)	48.4 ± 3.51	41.6 ± 3.91	40.2 ± 1.87
TSH (mU/L)	0.04 ± 0.007	0.04 ± 0.01	0.02 ± 0.007*
Heart rate (bpm)	99.8 ± 3.01	96.73 ± 2.80	87.2 ± 5.3^∗,∗∗^
Blood pressure (mmHg)	133.7 ± 2.19/77.6 ± 1.14	128.9 ± 2.01/77.4 ± 1.35	130.0 ± 2.8/78.0 ± 5.6

*P < 0.01 compared to the patients with TT genotype.

**P < 0.01 compared to the patients with AT and TT genotypes.

**Table 3:** Duration of disease in patients with Graves' disease, type 2 deiodinase gene Thr92Ala polymorphism genotypes.

	D2 gene genotypes			*P*
	T/T (*n* = 106)	A/T (*n* = 60)	A/A (*n* = 14)	
Total duration of the disease (months)	32.2 (7.9; 131.8)	16.8 (4.2; 67.1)	14.0 (5.6; 35.2)	0.007
Duration of overt thyrotoxicosis (months)	15.5 (5.9; 40.6)	10.9 (4.8; 25.0)	10.0 (4.2; 24.1)	0.04

**Table 4:** Remission and recurrence frequencies in patients with Graves' disease, type 2 deiodinase gene Thr92Ala polymorphism genotypes.

	D2 gene genotypes		
	TT (*n* = 52)	TA (*n* = 30)	AA (*n* = 13)
Recurrence	65.4%	60.0%	15.4%
Remission	34.6%	40.0%	84.6%
